# Supplementary material for: Protective Effects of a Jellyfish-Derived Thioredoxin Fused with Cell-Penetrating Peptide TAT-PTD on H2O2-Induced Oxidative Damage
Source: Int J Mol Sci. 2023 Apr 16;24(8):7340. doi: 10.3390/ijms24087340 (PMC10138494; doi:10.3390/ijms24087340)
Supplement: Supplementary file 1 [file ijms-24-07340-s001.zip › Supplementary material-Figure S1.pdf]

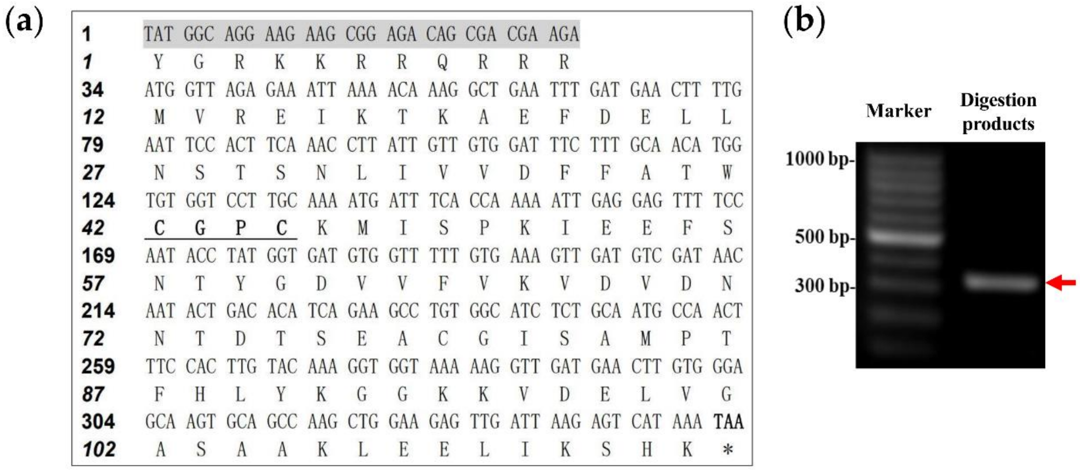

Figure S1. Sequence analysis and identification of PTD-CcTrx1 coding gene. (**a**) The full-length cDNA nucleotide and deduced amino acid sequences of PTD-CcTrx1. The sequence of PTD coding gene is marked in grey. The characteristic active site ($_{42}$CGPC$_{45}$) is bold and underlined. (**b**) Nucleic acid electrophoresis of the digestion products of PTD-CcTrx1 recombinant plasmid. The position corresponding to the inserted PTD-CcTrx1 coding sequence is indicated by the red arrow.
