# Supplementary figures and images for: Protective Effects of a Jellyfish-Derived Thioredoxin Fused with Cell-Penetrating Peptide TAT-PTD on H2O2-Induced Oxidative Damage
Source: Int J Mol Sci. 2023 Apr 16;24(8):7340. doi: 10.3390/ijms24087340 (PMC10138494; doi:10.3390/ijms24087340)

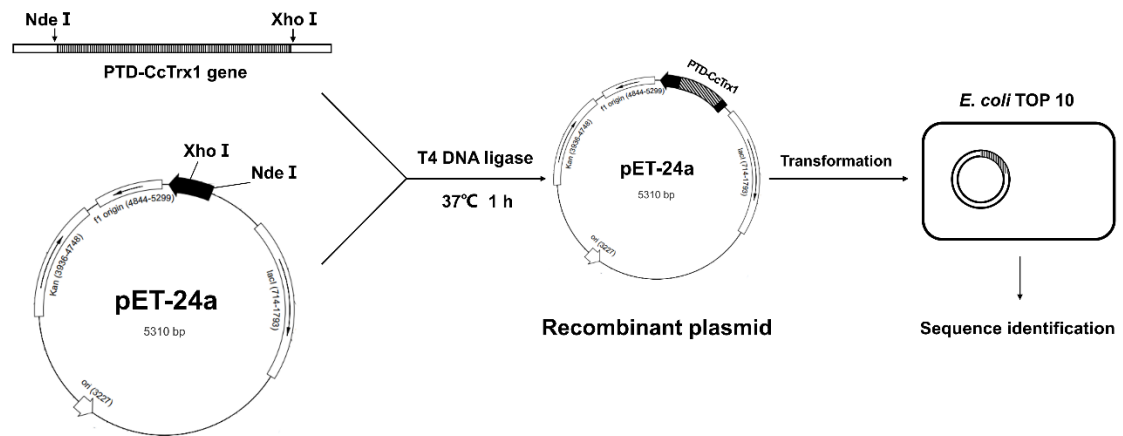

**Figure S2.** Construction of PTD-CcTrx1 expression vector.

Supplement: Supplementary file 1 [file ijms-24-07340-s001.zip › Supplementary material-Figure S2.pdf]
